# Integrating local ecological knowledge into systematic conservation planning for seahorse conservation

**DOI:** 10.1111/cobi.70027

**Published:** 2025-05-30

**Authors:** Anna Karolina Martins Borges, Vanessa M. Adams, Rômulo Romeu Nóbrega Alves, Tacyana Pereira Ribeiro Oliveira

**Affiliations:** ^1^ Programa de Pós‐Graduação em Etnobiologia e Conservação da Natureza Universidade Federal Rural de Pernambuco Recife PE Brazil; ^2^ LAPEC ‐ Laboratório de Peixes e Conservação Marinha Universidade Estadual da Paraíba João Pessoa PB Brazil; ^3^ School of Geography, Planning, and Spatial Sciences University of Tasmania Hobart Tasmania Australia; ^4^ Centre for Marine Socioecology, Institute for Marine and Antarctic Studies, College of Sciences and Engineering University of Tasmania Hobart Tasmania Australia; ^5^ Departamento de Biologia Universidade Estadual da Paraíba Campina Grande PB Brazil; ^6^ Centro de Ciências Biológicas e Sociais Aplicadas Universidade Estadual da Paraíba João Pessoa PB Brazil; ^7^ Seahorse, Pipefish and Seadragon Specialist Group International Union for Conservation of Nature (IUCN) Species Survival Commission Gland Switzerland

**Keywords:** socioecological systems, conservation planning, Syngnathidae, Marxan, participatory mapping, ethnoecology, local ecological knowledge, conocimiento ecológico local, etnoecología, mapeo participativo, Marxan, planeación de la conservación, sistemas socio‐ecológicos, Syngnathidae, 社会生态系统, 保护规划, 海龙科(Syngnathidae), Marxan软件, 参与式绘图, 民族生态学, 当地生态知识

## Abstract

Successful long‐term conservation relies on strategic planning for pragmatic actions to mitigate threats. Prioritizing actions and areas to support conservation goals in the most cost‐effective scenario becomes crucial in resource‐limited environments. However, planning and management can be challenging in data‐limited contexts. Incorporating local ecological knowledge (LEK) into conservation planning is an underexplored method of addressing these knowledge gaps. We utilized systematic conservation planning to identify key sites for seahorse threat management in a complex social‐ecological system in a protected area. Through participatory mapping and interviews with artisanal fishers, we gathered insights about seahorses, threats to them, and their socioeconomic significance for the local community. We compared LEK‐derived seahorse conservation priorities with spatial priorities identified using Marxan and with LEK‐derived and science‐derived data to explore LEK's contribution to spatial planning for a data‐poor species and to explore different seahorse threat management scenarios. The LEK‐derived and science‐derived seahorse abundance Marxan scenarios had a strong spatial agreement, emphasizing LEK's role in conservation planning. Furthermore, LEK‐derived data filled key data gaps on the distribution and nature of water‐based threats. Threat management scenarios for land and water‐based threat management had distinct spatial patterns. Incorporating LEK into decision‐making empowered local communities and thus fostered community‐based management. These findings offer insights into conservation planning in data‐deficient scenarios and can aid decision makers and local stakeholders in inclusive conservation strategies. Our results identified priorities for seahorse conservation in the Rio Formoso Estuary and our methods offer a transferable approach for participatory and interdisciplinary planning, which are essential for biodiversity conservation and livelihoods maintenance.

## Abstract (es)

Integración del conocimiento ecológico local dentro la planeación sistemática de la conservación del caballito de mar

**Resumen:**

El éxito de la conservación a largo plazo depende de la planificación estratégica de acciones pragmáticas para mitigar las amenazas. En entornos con recursos limitados, es crucial priorizar las acciones y las zonas para apoyar los objetivos de conservación en el escenario más rentable. Sin embargo, la planificación y la gestión pueden resultar difíciles en contextos con escasez de datos. La incorporación del conocimiento ecológico local (CEL) a la planificación de la conservación es un método poco explorado para abordar estos vacíos de conocimiento. Utilizamos la planificación sistemática de la conservación para identificar lugares clave para la gestión de la amenaza del caballito de mar en un complejo sistema socio ecológico de un área protegida. Con la cartografía participativa y entrevistas a pescadores artesanales, recopilamos información sobre los caballitos de mar, sus amenazas y su importancia socioeconómica para la comunidad local. Comparamos las prioridades de conservación de los caballitos de mar derivadas del CEL con las prioridades espaciales identificadas mediante Marxan y con los datos derivados del CEL y los derivados de la ciencia para explorar la contribución del CEL a la planificación espacial de una especie con pocos datos y para explorar diferentes escenarios de gestión de las amenazas a los caballitos de mar. Los escenarios de Marxan de abundancia de caballitos de mar derivados del CEL y derivados de la ciencia tuvieron una fuerte concordancia espacial, enfatizando el papel del CEL en la planificación de la conservación. Además, los datos derivados del CEL llenaron vacíos de información clave sobre la distribución y naturaleza de las amenazas de origen acuático. Los escenarios de gestión de amenazas terrestres y acuáticas presentaron patrones espaciales distintos. La incorporación del CEL mejoró la toma de decisiones y empoderó a las comunidades locales, lo que fomenta la gestión comunitaria. Estos resultados ofrecen una visión de la planificación de la conservación en escenarios con escasez de datos y pueden ayudar a los responsables de la toma de decisiones y a los actores locales a definir estrategias de conservación inclusivas. Nuestros resultados identificaron prioridades para la conservación del caballito de mar en el estuario del río Formoso y nuestros métodos ofrecen un enfoque transferible para la planificación participativa e interdisciplinaria, que son esenciales para la conservación de la biodiversidad y el mantenimiento de los medios de subsistencia.

## 将当地生态知识纳入海马保护的系统性保护规划

**【摘要】** 长期保护的成功有赖于对减缓威胁的实际行动进行战略规划。在资源有限的环境中, 以最具成本效益的方案确定能够支持保护目标的优先行动及优先区域至关重要。然而, 在数据有限的情况下, 保护规划和管理会面临挑战。将当地生态知识(local ecological knowledge, LEK)纳入保护规划是弥补这些知识空缺的一种有待探索的方法。本研究利用系统性保护规划, 在一个保护地的复杂社会生态系统中确定了海马威胁管理的关键地点。通过参与式绘图和对个体渔民的访谈, 我们收集了有关海马、其面临威胁及其对当地社区的社会经济意义的看法。我们将利用LEK确定的海马优先保护规划与Marxan软件确定的空间优先保护区, 以及基于LEK的数据和科学数据进行了比较, 以探究LEK如何帮助一个缺乏数据的物种进行空间规划, 并探索了不同的海马威胁管理方案。我们发现, 基于LEK和基于科学数据的海马丰度Marxan情景在空间上具有很强的一致性, 表明LEK在保护规划中具有重要作用。此外, 来自LEK的数据为有关水环境威胁分布和性质的关键数据填补了空缺。陆地环境和水环境威胁管理方案具有不同的空间格局。纳入LEK有助于决策和增强当地社区的能力, 从而促进基于社区的管理。这些发现为数据不足情况下的保护规划提供了见解, 可以帮助决策者和当地利益相关者制定包容性的保护战略。我们的研究结果确定了里约福莫索河口海马保护的优先规划, 本研究还为参与式规划和跨学科规划提供了一种可推广的方法, 这对生物多样性保护和生计维持至关重要。**【翻译:胡怡思;审校:聂永刚】**

## INTRODUCTION

Marine and coastal ecosystems are confronted with a multitude of threats arising from human activities on land and at sea. These threats will persist and intensify unless appropriate management actions are implemented (Halpern et al., 2015). The creation of well‐managed marine protected areas (MPAs) has been demonstrated as an effective approach to abating threats and ultimately protecting biodiversity (Pendleton et al., 2018). An important aspect of establishing MPAs is therefore identifying conservation features of interest, their spatial distribution, and subsequent prioritization of areas to protect them. Systematic conservation planning (SCP), an approach founded on well‐defined objectives, can aid in the identification, arrangement, and management of marine areas (Margules & Pressey, 2000). This approach enables the design of networks of protected areas and conservation strategies that meet quantitative targets through the use of optimization tools to determine the most efficient and effective spatial configurations (Ball et al., 2009).

Although SCP is considered the best practice in the spatial design of conservation actions, it relies on appropriate spatial data, such as species distribution, habitat types, and resource user patterns being available for the planning region (Pressey & Bottrill, 2009). Historically, these data have been sourced from databases containing, for example, species occurrence records, or remotely sensed data and combined with modeling approaches to meet decision support tool requirements, such as continuous data layers. We termed this type of data used in SCP *science‐derived data*. The extent and coverage of science‐derived data are often limited in marine environments due to high costs and logistical challenges of data collection and processing into appropriate spatial layers (Ban, 2009).

Local ecological knowledge (LEK), defined here as “a comprehensive system encompassing the understanding, practices, and insights about ecosystems, species, and ecological dynamics held by individuals living in close association with natural environments” (Davis & Wagner, 2003; Gerhardinger et al., 2009), has been increasingly used to confront the challenge of limited data to support the design and management of MPAs. For example, it has been employed to develop spatially explicit maps of threats (Noble et al., 2020), priority areas for iconic species (Noble et al., 2020), and data‐deficient marine habitats (Teixeira et al., 2013).

Marine and coastal ecosystems represent complex socioecological systems, embodying a blend of cultural, economic, historical, and political influences and hosting multiple actors with diverse perspectives and conflicting goals (Rivers et al., 2023). Effective conservation planning in these environments requires frameworks that recognize the interconnectedness between humans and the natural world and design management actions appropriately (Ban et al., 2013; Guerrero & Wilson, 2017). Incorporating LEK into SCP can thus serve a dual purpose: addressing data gaps and enhancing decision‐making by fostering the cocreation of knowledge, engaging and empowering local communities, and developing management solutions that support a diverse range of socioecological priorities (Ban et al., 2013; Noble et al., 2020).

Marine and coastal environments are biodiverse ecosystems; however, species in these environments are often poorly described or data deficient (Appeltans et al., 2012). Data‐deficient species are likely to be threatened and may require species‐level expert‐derived conservation strategies to address data gaps (Borgelt et al., 2022). For example, seahorses (*Hippocampus* spp.) are rare and data‐poor marine fishes and particularly susceptible to human‐derived activities in coastal environments (Zhang & Vincent, 2019b). Among the 46 recognized species of seahorses, 14 are classified as threatened (two endangered and 12 vulnerable) and 17 are classified as data deficient (IUCN, 2022). Planning for the conservation of seahorses is a key strategy as part of a broader effort to safeguard biodiversity and its dependent communities (Vincent et al., 2011). Despite the urgency, there has been only one prioritization study for seahorses, which was conducted at a regional scale in China and on a global scale (Zhang & Vincent, 2019a). In Brazil the three recognized seahorse species are nationally recognized as vulnerable (MMA, 2022); however, to date there are no species‐specific conservation plans in place for seahorses, and their broader conservation in MPAs has not been assessed.

As a globally significant megadiverse country, Brazil has achieved substantial advancement in establishing protected areas (Maretti et al., 2019). However, like many countries, Brazil faces challenges in managing effective and equitable MPAs (Giraldi‐Costa et al., 2020). Efforts in Brazil over the past two decades have aimed to incorporate society into the management of protected areas by introducing participatory guidelines and frameworks into legislation (Lima et al., 2021). Nevertheless, the creation and management of MPAs in Brazil are predominantly carried out through top‐down planning approaches reliant on science‐derived data and often overlook or marginally consider the involvement of local stakeholders (Bockstael et al., 2016; Gerhardinger et al., 2009). This risks creating paper parks that are not effectively managed and thus do not protect the species of interest (Mills et al., 2020). Furthermore, for coastal species, such as seahorses, relying solely on protected areas, even when effectively enforced, may not provide sufficient protection due to threats extending beyond these zones (Yasué et al., 2012). Site‐specific data on threats and spatially explicit threat management are essential to ensure threats are abated in‐ and outside MPAs (Mazaris et al., 2019). However, there are commonly gaps in science‐derived data for coastal and marine species; thus, additional complementary data, such as LEK, are essential.

We used seahorses and a complex social‐ecological system in the Rio Formoso Estuary as a case study to explore the LEK contribution to spatial planning for managing threats. Our overarching aim was to answer the question, where should efforts and resources be directed to guarantee pragmatic and effective conservation actions? To answer this, we collected LEK and science‐derived data suitable for use in the conservation prioritization tool Marxan and explored spatial prioritizations based on the different data sources. We compared these spatial priorities to explore LEK's contribution to supporting spatial planning for data poor species and examine how spatial priorities vary for different threat management scenarios. By doing so, we expected to glean valuable insights into strategies for conservation planning in data‐poor contexts and LEK integration that would assist decision‐makers and local stakeholders in devising adaptable and participatory solutions that harmonize social and ecological imperatives.

## METHODS

### Study area

**FIGURE 1:**
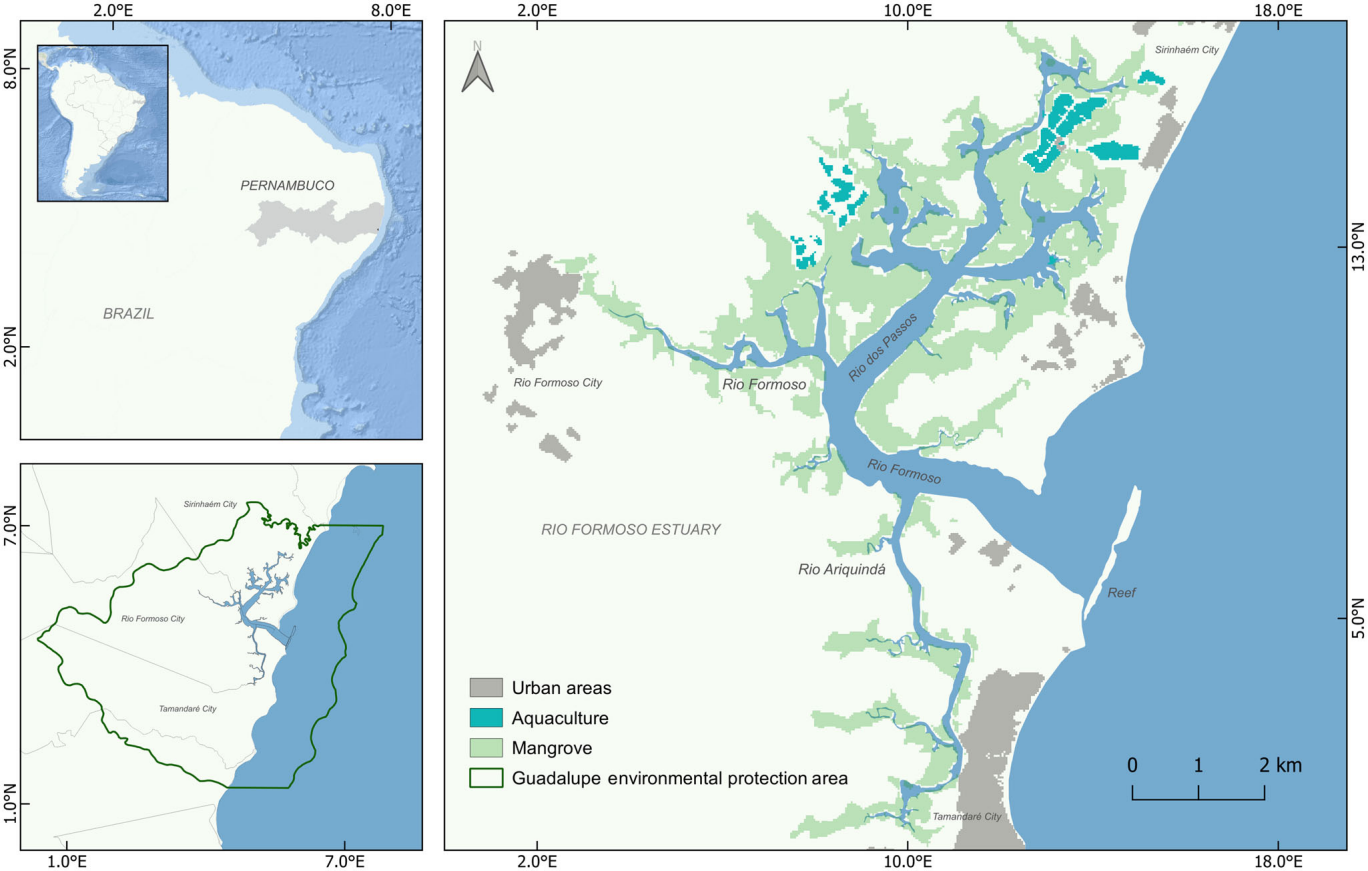
Location of the Rio Formoso Estuary in the Guadalupe Environmental Protection Area (Pernambuco State, Brazil).

The study took place in the Rio Formoso Estuary, south coast of Pernambuco state, northeastern Brazil (Figure 1). The estuary covers approximately 28 km^2^ and contains three main rivers and extensive mangrove forests. Rio Formoso Estuary is recognized for its human and ecological landscape values (CPRH, 2011; Silva et al., 2009, 2003). It is in the Guadalupe Environmental Protection Area (Área de Proteção Ambiental de Guadalupe—APAG), a sustainable‐use MPA. This MPA category seeks to provide natural resource conservation and to maintain environmental quality for local communities through management plans and zoning that allows several forms of human use (MMA, 2000; Rylands & Brandon, 2005). The APAG faces several socioenvironmental conflicts, primarily among user groups, including tourism, nonselective fishing, shrimp farming, and agriculture (Araújo et al., 2014; Santos, 2002). Many of these are considered major threats to the longsnout seahorse (*Hippocampus reidi* Ginsburg, 1933), which occurs in the estuary (Aylesworth et al., 2015; Borges et al., 2023).

**FIGURE 2:**
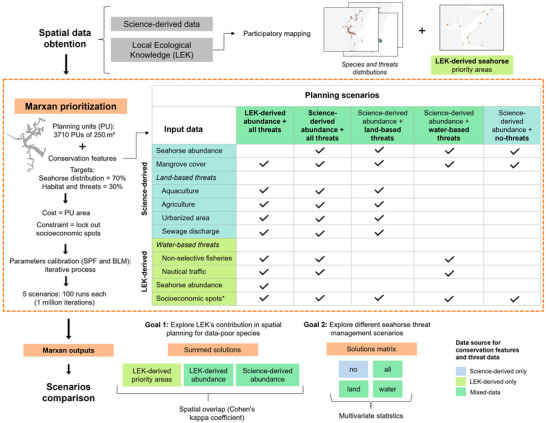
The methodological steps and data types used in conservation spatial prioritization and the comparison of various scenarios for threat management for seahorses. Detailed descriptions of each dataset are in Appendix S3 (*, socioeconomic spots layer used to lock out planning units with socioeconomic importance for the local community; SPF, species penalty factor; BLM, boundary length modifier).

In our study area, the primary conservation planning problem is where to deploy limited resources to manage threats to seahorse populations and habitat in the existing designated sustainable‐use MPA. As a result of discussions with local stakeholders, we grouped threats to seahorses into two main groups: land‐based threats (diffuse pollution from agriculture, aquaculture, and urban areas and point source pollution from sewage discharge) and water‐based threats (nonselective fishing and nautical traffic). Therefore, we sought to identify conservation targets for seahorses and coastal habitat (mangroves) that overlapped with land‐based and water‐based threats in a minimum amount of area (to minimize operational costs for local managers) and avoided areas that were socioeconomically important to local communities (to minimize potential conflicts). To this end, we adopted an SCP approach in which the first step was to identify available (science‐derived) data aligned with our conservation objectives and to then fill gaps with relevant methods (in our case LEK) (Pressey & Bottrill, 2009) (Figure 2).

### Reviewing and compiling science‐derived data

We compiled academic spatial data on seahorse distribution, habitats, and threats and ensured that the data met the requirements for use in spatial prioritization, namely that the spatial layer was complete across the study region. This resulted in two science‐derived layers representing conservation targets: seahorse abundance and mangrove extent (Figure 2). Because seahorses are the primary focus of this conservation plan, we used the best available science‐derived layer for seahorse abundance from Borges et al. (2023). This layer is based on academic under water visual survey transects processed into a continuous map of abundance with a kernel density function (see Appendix S3 for full details and map). The secondary conservation feature for this study was mangroves because they are the most important habitat for seahorses (Borges et al., 2023). The spatial layer for mangrove cover was used (available from the sixth collection of the open‐source MapBiomas Project, https://mapbiomas.org/). Finally, we considered possible sources of data for threats. We used land use data from MapBiomas as surrogates for the presence of point source pollution from untreated sewage discharge and the presence of diffuse pollution from agriculture, aquaculture, and urban areas (see Appendix S3 for full methods and figures). This compilation of available science‐derived data identified key gaps in data are needed to support spatial planning, namely water‐based threats. Therefore, the next step was to complement these data with LEK.

### Accessing LEK

Due to the lack of data available for the area, we elicited LEK from small‐scale fishers and boat operators actively residing and working in the estuary. We focused on gathering insights into seahorse ecology, threats, and potential management strategies (Figure 2, LEK‐derived data). For that we applied a mixed‐method approach that encompassed participatory mapping exercises (with individuals and in focus groups; Leis et al., 2019) and semistructured interviews (Albuquerque et al., 2014; Huntington, 2000).

To elicit spatial data on the location of priority species and threats, we recruited artisanal fishers to participate in the semistructured interviews. Participants were intentionally chosen using a criterion sampling method (Bryman, 2016) combined with snowball sampling (Bailey, 1994; Naderifar et al., 2017). We first identified key informants with a leadership role in the community. These informants recommended other potential participants based on their status as local experts in possession of extensive knowledge of both the estuary and seahorses. When repetition emerged in recommendations, we considered that saturation had been reached. We identified 25 potential participants, and they all willingly consented to participate (participant demographics in Appendix S1). Interviews were structured, and the questionnaire covered participants’ socioeconomic background, their knowledge about the study's focal species (e.g., occurrence, habitat, exploitation, and threats), and their perceptions and attitudes concerning seahorse conservation in the estuary that encompassed biological and socioeconomic impacts (questionnaire in Appendix S2).

Following the interviews, fishers were provided with a basic map of the estuary, illustrating territorial boundaries, the coastline, and rivers (Leis et al., 2019; Noble et al., 2020). They were then instructed to mark on the map significant fishing locations and areas relevant to seahorse conservation planning. This included aspects such as seahorse occurrence and habitat areas, points of conflict with activities affecting seahorses, and regions of economic and sociocultural significance. Additionally, they were prompted to mark on the map locations they deemed significant for directing efforts toward seahorse protection, thus designating priority areas. Their selections were guided by their understanding of seahorse distribution and potential threats. The interviews were done in person and audio recorded. Data collection was conducted under human ethics approval by the Universidade Federal Rural de Pernambuco's Human Research Ethics Committee (protocol 4.807.374).

To complement the interviews, we organized workshops with a wide cross‐section of estuary user groups. In December 2021, two workshops were conducted in municipalities in the protected area (Tamandaré and Rio Formoso). To identify participants, we collaborated with community organizations, including the fishers’ association, boat marina, and the protected area administration. The organizations recommended key participants who volunteered to take part in the workshops. In total, 24 local stakeholders participated, spanning a variety of groups, including small‐scale fishers, boat operators, entrepreneurs, protected area managers, and environmental government agencies (workshop participant details in Appendix S1). The workshops followed a structured process, consisting of three key phases: first, introduction in which the workshop facilitator outlined the main objective of the workshops; second, participatory mapping in which participants’ engaged in focus groups to discuss and indicate on large maps the areas or specific locations in the estuary where threats to seahorses were identified, the nature of the threat, and linked management options; and third, discussion and brainstorming in which the facilitator synthesized the mapped information and encouraged participants to contribute key insights regarding seahorse threats and potential management solutions to address these threats.

### Collating spatial data on conservation features and threats from LEK

The data obtained from the participatory mapping exercises were digitized and analyzed using QGIS 3.32.2. Based on the participatory mapping, we sought to determine seahorse conservation priorities based solely on LEK‐derived data. To this end, we generated a map of priority areas for seahorse conservation according to the fishers (LEK‐derived seahorse priorities, Figure 2; full details in Appendix S5).

We further used the LEK data to derive spatial layers for use in spatial prioritization (with Marxan) for the following data: seahorse abundance, water‐based threats, and areas of socioeconomic importance (Figure 2) (full details in Appendix S3). For use in Marxan, the LEK‐derived abundance of seahorse was determined based on a count of mentions in each planning unit. The LEK‐derived water‐based threat distribution data were converted into a spatial layer of threat presence to match the data format for land‐based threats. Further details on the construction of each layer are in Appendix S3.

### Conservation planning scenarios

Our overarching aim was to identify priority areas for threat management to conserve seahorses and to do so in a way that is cost‐effective and stakeholder inclusive. Related to this aim and conservation objectives (targeting seahorse abundance for threat management) we defined two research goals and associated conservation planning scenarios. The first goal was broadly to identify the influence of sources of data for seahorse abundance (LEK or science‐derived data), and the second was to consider threat data that could guide management activities (on land, on water, or on both land and water). A summary of the scenarios and conservation features considered in each scenario is in Figure 2.

To address our first goal of exploring LEK's contribution to spatial planning for data poor species, we compared LEK‐derived spatial priorities with those from Marxan. We further defined two Marxan scenarios that differed only in the source of seahorse abundance data used (LEK‐derived or science‐derived abundance map). We named these scenarios based on the source of seahorse data: LEK‐derived abundance and science‐derived abundance.

Our second goal was to explore seahorse threat management scenarios. To do so, we considered four Marxan scenarios in which the primary conservation features were the science‐derived seahorse abundance and the type of threat included. Threat types were all threats (this is the same Marxan run as the science‐derived abundance scenario for our first goal), land‐based threats, water‐based threats, and no threats (Figure 2).

### Marxan objectives and conservation targets

To identify spatial priorities for each scenario, we employed the decision support tool Marxan via the CLUZ interface (http://www.kent.ac.uk/dice/cluz/) within QGIS. Marxan is one of the most widely used prioritization tools for SCP (Ball et al., 2009). We partitioned the study area into 3710 hexagonal PUs with a maximum size of 250 m^2^ (based on the input data resolution).

To match our conservation objective, we identified two conservation features of interest: the seahorse species *H. reidi* and its mangrove habitat. We set a conservation target of protecting 70% of the distribution area or population size (here, measured by abundance) of *H. reidi*. The target was based on the threat status and associated IUCN Red List criteria recommendations (IUCN, 2022). We set a target of 30% for mangrove cover. This target was selected as a midpoint within the recommended range of 20–50% (O'Leary et al., 2016). Because one of our objectives was to identify areas for threat management, threats were considered as targets in our planning scenarios, and we set a target of 30% for each threat distribution.

Considering the limited resources of the protected area, it is important to consider the costs associated with conservation plans. In our study region, there was an absence of spatially heterogeneous cost data. Therefore, we assumed that minimizing area was a reasonable approach to ensuring that areas selected fit within staff constraints, and we set planning unit cost equal to area. We further considered socioeconomic values via stakeholder participation in LEK (Ban et al., 2009; Possingham et al., 2000). To minimize impacts on stakeholders, we locked out areas of socioeconomic importance for the local community (elicited via the LEK workshops).

### Running spatial prioritization

For each conservation planning scenario (Figure 2), we ran Marxan 100 times with 100 million iterations. Marxan incorporates two weighting factors, the species penalty factor (SPF) and boundary length modifier (BLM), to strike a balance between conservation targets and total boundary length (Serra et al., 2020). We employed an iterative process to determine suitable values for SPFs (to ensure targets are met) and BLMs (to allow specified levels of spatial clumping) and ensure that our conservation goals were consistently achieved across all replicates while maintaining compact and cost‐effective priorities (Zhang & Vincent, 2019a; Appendix S4). For each scenario, we recorded run data, including total cost of the solution, number of selected PU's, total boundary length, Marxan penalty value, and number of conservation features that did not achieve targets in the solution. To compare scenarios, we used the run data recorded and the selection frequency (calculated the as the number of times out of 100 runs a planning unit was selected) to compare spatial overlaps in scenarios.

### Comparing scenarios

To explore LEK's contribution to spatial planning for data‐poor species, we compared the LEK‐derived seahorse priority areas with those identified using Marxan with the two sources of seahorse data (LEK‐derived distribution and science‐derived distribution) (goal 1, Figure 2). We used Cohen's kappa coefficient to verify level of spatial agreement between the stakeholder frequency mention (LEK‐derived seahorse priorities) and selection frequency for the two Marxan scenarios (kappa2 in R package irr [Gamer et al., 2019]) (Teschke et al., 2022). For this calculation, we classified selection frequency into five classes (0%, <25%, 25–50%, 50–75%, and >75%), as proposed by Ruiz‐Frau et al. (2015). Cohen's kappa can range from +1, indicating complete agreement, to −1 indicating complete disagreement (*k* = 0, overlap due to chance; *k* < 0.4, poor agreement; 0.4 < *k* < 0.75, good agreement; *k* > 0.75, excellent agreement) (Landis & Koch, 1977).

We compared the four threat management planning scenarios: all threats, land‐based threats, water‐based threats, and no threats (goal 2, Figure 2). To compare the solutions under the four threat management scenarios, we used individual run data in a hierarchical cluster analyses and nonmetric multidimensional scaling (nMDS) ordination, based on a Jaccard resemblance matrix. The analyses followed the recommendations and code amended from Harris et al. (2014) and were performed using the functions hclust, metaMDS, and vegdist in the Vegan package (Oksanen et al., 2020). A hierarchical cluster analysis dendrogram was constructed using the ColorDendrogram function of the sparcl package (Witten & Tibshirani, 2018). We applied an envfit analysis to determine which explanatory variables were best correlated across the nMDS ordinations. These variables included the individual run data as detailed above. Variables that showed significant correlations (*α* = 0.05) were then represented as axes on the bidimensional nMDS diagram. All analyses were performed in R 4.3.1 (R Development Core Team, 2023).

## RESULTS

Based on LEK and academic data, we constructed two maps of seahorse distribution and six threat maps (Figure 2 & Appendix S3). Linked to the threats, based on local stakeholder interviews and focus groups (fishers and boat operators), we identified 15 related threatening activities and associated management actions (Table 1). Management actions spanned place based (e.g., monitoring strategies) to generic measures (e.g., promoting awareness).

**TABLE 1:** Summary of threats and respective management actions identified by local stakeholders for seahorse conservation in the Rio Formoso Estuary.

Threat category	Threatening activity	Management action	
		Place‐based measures	Generic measures
Water‐based: nonselective fishing	Fishing net with small mesh	Size monitoring	Promote fishers’ awareness
	Fishing with explosives	Monitoring and punishment	Promote fishers’ awareness
	Fishing with poison (tickicide)	Monitoring and punishment	Promote fishers’ awareness
		Product sales control	
	Lack of attention to bycatch seahorses		Promote fishers’ awareness about the importance of removal and return
	Capture for medicinal purposes	Monitoring or punishment	Encouraging the use of alternative treatments
	Capture for ornamental purposes (aquarium)	Monitoring or punishment	Promote fishers’ awareness
	Mangrove deforestation	Monitoring or punishment	Promote fishers’ awareness
Water‐based: tourism and nautical traffic	Wakes created by exceeding speed limits (affect seahorses and mangroves)	Speed monitoring system	Promote boat operators’ awareness
		Installation of speed limit signs	
		Limit distance from riverbank according to vessel size or engine capacity	
	Seahorse capture for tourism display	Monitoring or punishment	Promote tourist and tourism operators’ awareness
	Navigation with engine on the river branches	Monitoring or punishment	Promote boat operators’ awareness
	Oil leakage from vessels	Speed limits according to engine capacity	Promote boat operators’ awareness
Land‐based: pollution	Pesticides and herbicides	Monitoring	Promote awareness
	Shrimp farming water discharges	Monitor cleaning product usage	
	Untreated sewage discharge		Call for sanitation measures
	Improper waste disposal	Monitoring or punishment	Reward for proper disposal

**FIGURE 3:**
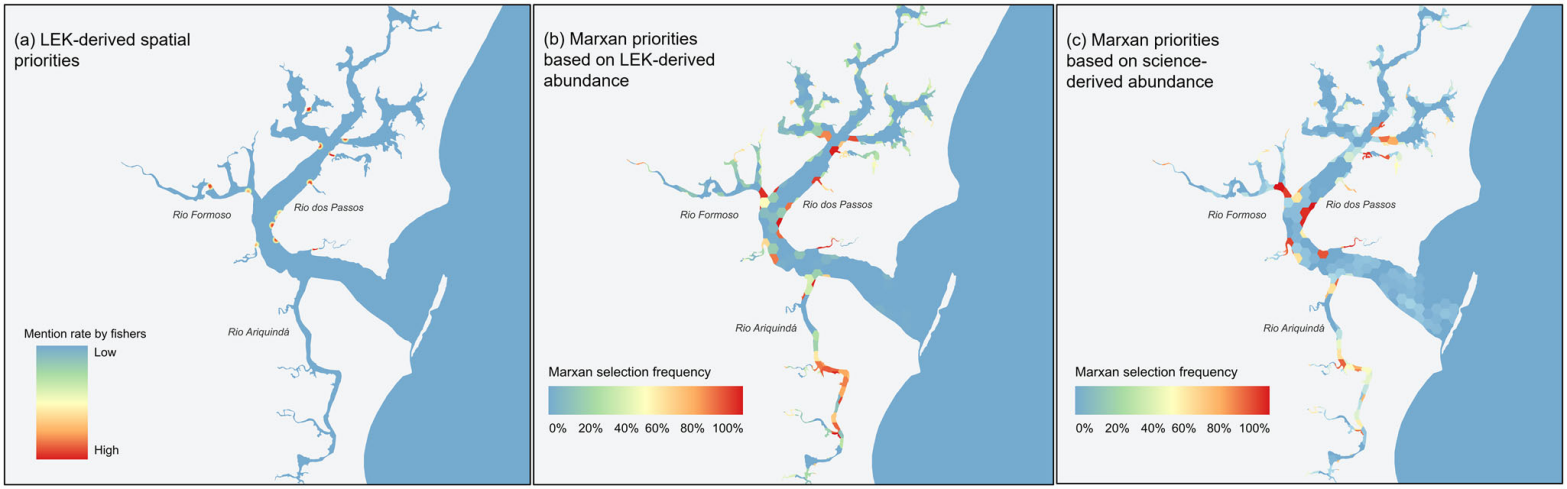
Priority conservation areas identified based on three planning scenarios to explore the contribution of LEK to threat management for seahorses: (a) areas identified by stakeholders (priorities based on mention rate by fishers), (b) Marxan scenario integrating local ecological knowledge (LEK)‐derived seahorse abundance data (priority areas mapped based on selection frequency), and (c) Marxan scenario with science‐derived seahorse abundance (priority areas mapped based on selection frequency).

For our first research goal, the spatial priority maps across the three scenario s (LEK‐derived spatial priorities and Marxan priorities based on LEK‐derived and science‐derived abundance) shared commonalities in spatial priorities (Figure 3). By visual inspection, we identified areas along the Rio dos Passos and Rio Formoso as priorities in all three scenarios (Figure 3). When comparing the statistical spatial agreement, the Marxan LEK‐derived scenario had excellent spatial agreement with the Marxan science‐derived scenario (*k* = 0.82). However, Cohen's kappa indicated poor spatial agreement between the LEK‐derived seahorse priorities and the Marxan based scenarios (*k* = 0.02) (Table 2). Visual differences that emphasized this were in Rio Ariquindá, where the Marxan scenarios selected priority areas and the LEK‐derived seahorse priorities scenario did not (Figure 3).

**TABLE 2:** Spatial similarity matrix comparing the selection frequency of planning units for the three scenarios of priority areas for threat management to conserve seahorses based on local ecological knowledge (LEK).^a^

Scenario^b^	LEK‐derived priority areas	Marxan LEK‐derived abundance
LEK‐derived priority areas	–	
Marxan LEK‐derived abundance	0.02	–
Marxan science‐derived abundance	0.02	0.82

^a^ Values are Cohen's kappa coefficient (range: −1 to +1) expressing agreement between the spatial solutions (*k* = 0, overlap due to chance; *k* < 0.4, poor agreement; 0.4 < *k* < 0.75, good agreement; *k* > 0.75, excellent agreement).

**FIGURE 4:**
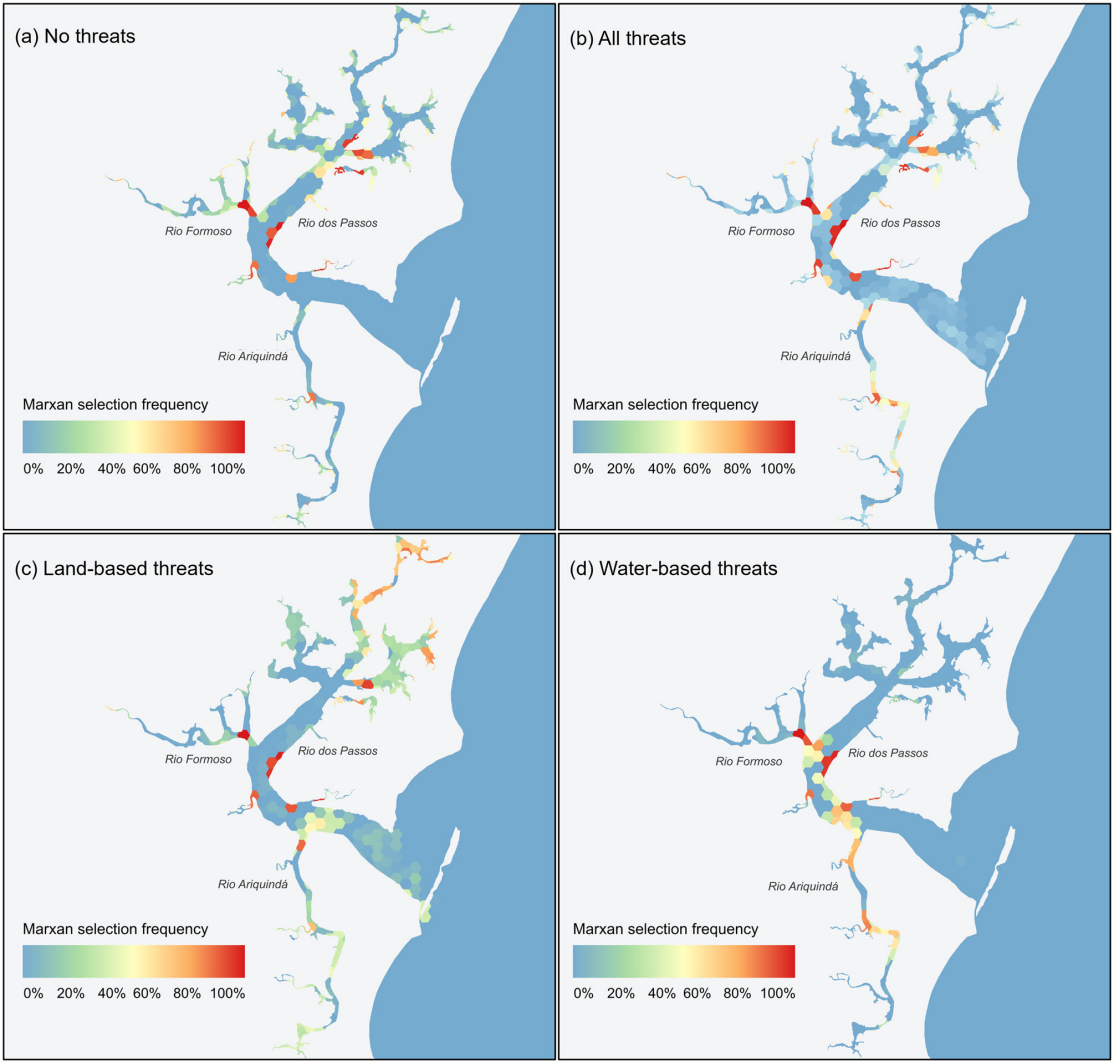
Marxan selection frequency (percentage of 100 runs) of conservation planning units for seahorses in the Rio Formoso Estuary for four scenarios of threat management targeting abundance: (a) no threats, (b) all threats (land and water based), (c) land‐based threats, and (c) water‐based threats.

In the four Marxan threat management scenarios, there were key differences in the patterns of spatial priorities, depending on the location of threats targeted (Figure 4). The scenarios in which all threats and no threats were considered had consistent patterns in areas frequently selected (>80%) (Figure 4a,b). In contrast, in the water‐based threats scenario, high selection frequencies concentrated along the rivers Formoso and Ariquindá. For the land‐based threat scenario, selection frequencies were more evenly distributed across all three rivers and concentrated in headwaters adjacent to human uses (Figure 4c,d).

**FIGURE 5:**
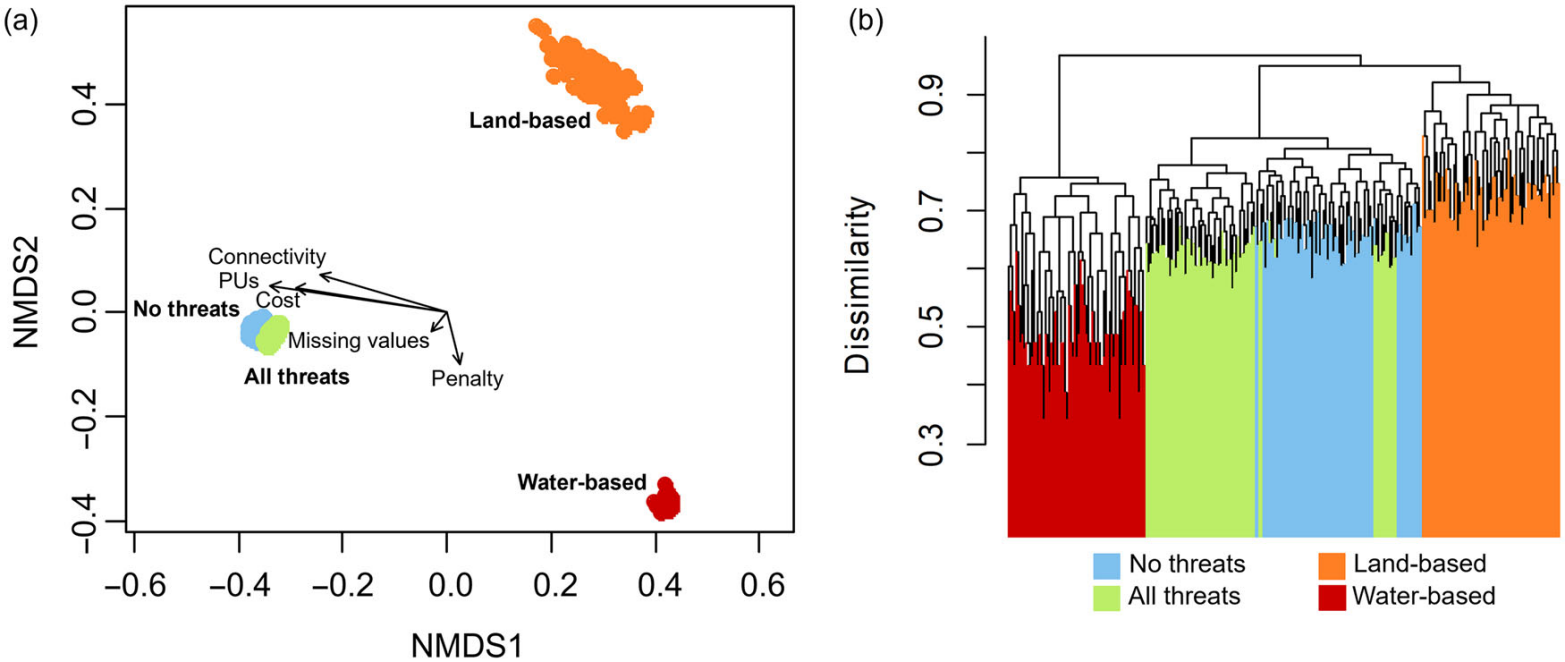
Spatial relationship among Marxan conservation planning solutions for each of the four scenarios for threat management for seahorses: (a) a nonmetric multidimensional scaling (nMDS) of the solutions based on a Jaccard resemblance matrix (vectors, significant model output parameters) and (b) results of the complete hierarchical cluster analysis (PUs, number of planning units selected in the reserve network; connectivity, reserve boundary length; penalty, Marxan penalty score; missing values, conservation features that did not meet targets in the solution).

The nMDS ordination showed statistically significant differences among the threat management solutions for the four scenarios (Figure 5). The scenarios with all threats and no threats were the most similar in their solutions. The scenarios with specific threats (land‐based only and water‐based only threats) were most dissimilar to the other scenarios (Figure 5). All vectors from the Marxan solution outputs were significantly correlated with the nMDS ordination structure (*p* < 0.05) (Figure 5), three of which had high correlation coefficients: number of PUs (*r* ^2^ = 0.987), cost (*r* ^2^ = 0.726), and connectivity (*r* ^2^ = 0.538). The Marxan penalty (*r* ^2^ = 0.087) and the missing values (*r* ^2^ = 0.018) were only weakly correlated with the nMDS surface.

## DISCUSSION

Our findings demonstrated the value of LEK as an important source of data to fill knowledge gaps in seahorse distribution and threats, which is in line with previous studies incorporating LEK into marine conservation planning (Ban et al., 2009; Noble et al., 2020). Although LEK is a critical and reliable data source, our results showed that relying solely on LEK to identify priorities may underestimate the required areas for meeting conservation targets (Figure 3). However, including LEK‐derived data significantly expanded our ability to consider threat distributions and associated management actions and thus provided a more nuanced understanding of where and how to conserve seahorses (Figure 4). Finding effective spatial solutions in data‐poor contexts represents a key challenge in SCP (Levin et al., 2014). In such instances, approaches that incorporate various data sources, such as LEK and academic data, become crucial in identifying the most adequate set of solutions. Our results demonstrated that the integration of LEK‐derived and science‐derived data with decision support tools (i.e., Marxan) offers a pragmatic and useful prioritization approach to identify conservation priorities in data‐poor contexts in regions characterized by numerous socioenvironmental conflicts.

### LEK informing seahorse distribution and threats

Dealing with data gaps in conservation planning is incredibly challenging in the context of areas with large territorial extensions and few resources available for conservation. This challenge is particularly pronounced in developing countries, such as Brazil, where limited capacity and resources are a prevalent decision‐making constraint (Weeks et al., 2015). Through LEK, we filled critical gaps in the knowledge of threats to seahorses in the Rio Formoso Estuary. Although our elicitation was specific to the Rio Formoso, the threats identified (land‐based pollution, nonselective fisheries, and nautical traffic from water‐based tourism) are aligned with the global understanding of threats to seahorse (Vincent et al., 2011) and mangroves (as the primary habitat for seahorses [Hamilton & Casey, 2016]). This alignment provides validity to LEK as a local source of knowledge on the types and distribution of threats to seahorses.

Water‐based threats can be challenging to define and observe using remote sensing data products. Therefore, LEK‐derived data were essential for defining and mapping water‐based threats in our study region. As reported by the local fishers, certain fishing practices employed in the estuary posed a significant threat to seahorses, involving intentional targeting and bycatch. Fishers attributed seahorse bycatch primarily to factors such as the use of small mesh sizes and the deployment of passive nets (e.g., gillnets or block nets; referred to as *camboa*) in close proximity to mangroves (termed nonselective fisheries [Vaidyanathan et al., 2021]) and mapped the distribution of such activities in the study region. Nautical activities resulting in boat‐generated wakes, in particular tourism, were the other primary water‐based threat identified by local fishers. In addition to affecting mangroves by causing erosion along the riverbanks (Huang et al., 2012), intense nautical traffic can directly affect seahorses (Bruto‐Costa, 2007). This problem is recognized in the estuary and has prompted the establishment of the environmental and territorial zoning of nautical activities (ZATAN); guidelines promote the sustainable management of watercourses in the Rio Formoso Estuary (SEMAS, 2021). The LEK mapped distribution of nautical activities can complement this zoning to further spatially target management.

The science‐derived maps indicated that most of the region is affected by land‐based pollution and that spatial patterns varied by activity (agriculture, aquaculture [shrimp farms], urban areas, and untreated sewage discharge) (Appendix S3). The middle portion of the estuary (mainly Rio dos Passos) is influenced by pollution, nutrients, and soil damage from aquaculture facilities focused on shrimp farming (Barcellos et al., 2019; Lacerda et al., 2021). Moreover, the estuary is surrounded by agricultural areas, predominantly sugar cane crops, where there is substantial usage of pesticides and herbicides (Pelage et al., 2023). Diffuse and point source pollution associated with urban areas (e.g., discharge of untreated sewage) are reported more for the upper estuarine zone (near Rio Formoso city). In a recent study, fishers reported similar threats to their fishing activities in the Rio Formoso Estuary (Pelage et al., 2023).

### Comparing LEK‐derived and science‐derived scenarios in spatial planning

Our findings demonstrated that incorporating LEK of data‐poor species distributions in SCP can be a valuable complementary data source to traditionally scientific data. The substantial spatial alignment between the LEK‐derived and science‐derived abundance Marxan scenarios highlighted the credibility and utility of incorporating LEK into spatial planning. Similar findings have been reported in other studies in which LEK was used to map the distribution of iconic marine species and habitats (Noble et al., 2020; Teixeira et al., 2013), to support conservation assessments (Leduc et al., 2021), and to gather crucial information to enhance fisheries management (Santos et al., 2019; Nunes et al., 2019).

The strong spatial agreement between Marxan scenarios, irrespective of the source of seahorse abundance data, emphasized the utility of LEK in mapping conservation features for use in SCP. However, our results for goal 1 showed that using LEK as the sole data could underestimate the extent of spatial priorities (Figure 3). The LEK‐derived seahorse priorities had poor spatial agreement with the Marxan solutions (Table 2), primarily due to the much smaller spatial extent of the areas mapped relative to those identified with Marxan (Figure 3). This result may reflect the constraints of the informants’ knowledge and potential socioeconomic conflicts in the planning region. For example, the LEK‐derived seahorse conservation priority map did not show any priority area in Rio Ariquindá. This river has been under high pressure due to intense boat traffic driven by tourism for decades, which hypothetically could also be related to tensions that lead fishers to avoid identifying these areas as conservation priorities (Araújo et al., 2014; Santos, 2002).

In contrast, it was clear through visual inspection that the most frequently selected areas in the LEK‐derived seahorse conservation priority map coincided with the Marxan scenario selection frequencies for both the LEK‐derived and science‐derived abundance scenarios (Figure 3). This is not a trivial finding, especially in situations where data are scarce and there is an urgent need to identify immediate conservation priorities for data‐poor species (Aylesworth et al., 2017; Thornton & Scheer, 2012). In these circumstances, decision makers and spatial planners can use LEK to gain initial insights for conservation priorities and expand these as more data are gathered and incorporated into SCP tools, such as Marxan (Game et al., 2011).

### Integrating LEK‐derived data in spatial prioritizations for threat management

Our research aim was to provide clear guidance to protected area management on where to focus their threat management efforts. Our findings underscore the important role of LEK in enhancing seahorse conservation efforts in the estuary. LEK offered a cost‐effective and participatory means to gather spatial data on threats and species distribution, particularly in data‐poor contexts. Insufficient quality, resolution, and coverage of spatial data can undermine the effectiveness of SCP (Ball et al., 2009; Ban & Klein, 2009; Mills et al., 2010). Fisher knowledge is increasingly recognized as a valuable data source for SCP initiatives, particularly in developing MPAs that garner community support while minimizing adverse impacts on fishers (Grantham et al., 2013; Teh et al., 2013; Yates & Schoeman, 2015).

The LEK‐derived threat data enabled us to consider planning scenarios for land and water‐based threats (Figure 4). The distinct spatial patterns in the scenarios emphasized the spatially distinct priority areas for different threat management actions. For instance, water‐based threats were concentrated along specific rivers, whereas land‐based threats exhibited a more dispersed distribution. These patterns can guide tailored conservation efforts based on the nature and location of the threat management, which can complement enforcement of protected areas as place‐based measures (Delaveux et al., 2019; Mazaris et al., 2019).

Our approach to eliciting LEK also considered stakeholder preferences for threat management strategies, which can guide the development of place‐based management plans to reduce or mitigate threats and improve conservation outcomes (Delaveux et al., 2019; Mazaris et al., 2019). The recommended strategies, as elicited from fishers (Table 1), can be drawn on when designing management plans in priority places. Such an approach to embedding LEK in the spatial prioritization and subsequent management strategy design has the potential to increase stakeholder support for conservation plans (Teh et al., 2013; Yates & Schoeman, 2015). These findings underscore the potential for participatory management, combining fishers’ knowledge with practical strategies, to address seahorse threats effectively (Knights et al., 2013). Beyond data provision, LEK integration plays a pivotal role in fostering a cocreative and participatory planning process, which can promote resource conservation and ecological well‐being (Dorrington et al., 2018).

### Place‐based recommendations for threat management and final considerations

Local stakeholders’ participation plays a significant role in SCP because it fosters inclusive decision‐making processes and enriches the data used for conservation strategies. The engagement of local communities is particularly important because it helps align conservation initiatives with socioeconomic priorities, strengthens the sense of stewardship among residents, and enhances the effectiveness of conservation measures in MPAs (Bennett & Dearden, 2014; Mills et al., 2020). Global (Cinner et al., 2012; Holness et al., 2022) and local examples (Campos‐Silva et al., 2017; Lima et al., 2021; Lopes et al., 2013) of bottom‐up and community‐based approaches to management have shown a pathway to reduce conflicts and deliver successful biodiversity and livelihood outcomes. However, challenges, such as continued governance through top‐down approaches, conflicting interests, and varying degrees of social cohesion among communities, often influence the extent and effectiveness of community involvement in MPA governance in Brazil (Gerhardinger et al., 2009; Mills et al., 2020). Integrating LEK into SCP to identify spatial priorities can foster local stakeholder participation (Scholz et al., 2004).

Enabling local stakeholders to participate in planning processes through a knowledge‐building approach presents a significant opportunity for MPAs to enhance management effectiveness (Gerhardinger et al., 2009; Mills et al., 2020; Karnad, 2022). In the Rio Formoso Estuary, local stakeholders indicated several place‐based management actions for seahorse conservation. These actions, linked to specific threats, ranged from site‐specific measures (e.g., speed monitoring for boat traffic) to broader measures (e.g., promote awareness) and underscore the significant and valuable contribution local stakeholders can make to problem‐solving in the management process. Those findings shed light on significant opportunities for the Guadalupe Environmental Protection Area, the goals of which extend beyond seahorse conservation to encompass broader conservation goals. These goals include considerations for revising the management plan through a participatory and inclusive process and reinforcing the boating zoning (ZATAN) implementation to address several of the threats pointed out by the local stakeholders. Leveraging the seahorse's status as a flagship species to reinforce management measures is another valuable opportunity for conservation in the study region (Vincent et al., 2011).

We harnessed the power of the Marxan software, integrating LEK and science‐derived data, to construct transparent, objective, and spatially efficient solutions for the management of threats in a situation where data were limited. Our results highlight the importance of interdisciplinary approaches, the integration of local knowledge, and the need for adaptive conservation strategies that consider the role of different threat management scenarios within the relevant social‐ecological context. The spatial outputs from our analyses and the management knowledge provided by LEK provide powerful tools to support seahorse conservation in the estuary. Moreover, the insights gained can guide collaborative planning and community‐based management to help secure biodiversity and livelihoods in this complex socioecological system.

## Supporting information



Supplementary Materials.
