# Supplementary material for: Integrating local ecological knowledge into systematic conservation planning for seahorse conservation
Source: Conserv Biol. 2025 May 30;39(3):e70027. doi: 10.1111/cobi.70027 (PMC12124176; doi:10.1111/cobi.70027)
Supplement: Supplementary file 1 — Supplementary Materials. [file COBI-39-e70027-s001.docx]

**Supplementary Material**

Integrating local ecological knowledge into systematic conservation planning for seahorse conservation

[S1. Selection and profile of study participants 0](file:///C:\Users\karol\Downloads\ddi13183-sup-0001-supinfo.doc#_Toc52261702)1

[S2. Questionnaire to access LEK 0](file:///C:\Users\karol\Downloads\ddi13183-sup-0001-supinfo.doc#_Toc52261702)2

[S3. Data used, processing, and details on conservation features mapping 03](file:///C:\Users\karol\Downloads\ddi13183-sup-0001-supinfo.doc#_Toc52261703)

[S4. Marxan parameters calibration 06](file:///C:\Users\karol\Downloads\ddi13183-sup-0001-supinfo.doc#_Toc52261703)

[S5. LEK-Derived spatial priorities 06](file:///C:\Users\karol\Downloads\ddi13183-sup-0001-supinfo.doc#_Toc52261703)

[REFERENCES 11](file:///C:\Users\karol\Downloads\ddi13183-sup-0001-supinfo.doc#_Toc52261707)

**S1. Selection and profile of study participants**

*Interviews and participatory mapping*

We carry out semi-structured interviews and participatory mapping to elicit spatial data on the location of priority species and threats. For this stage of the study, we recruited artisanal fishers and shellfish collectors (mainly women) who live and work in the municipality of Rio Formoso, deliberately selected due to their status as "local experts”, possessing extensive knowledge of both the estuary and seahorses. Details about the sampling methodology are described in main text. Using these criteria and sampling, we identified and reached out to all the indicated individuals as "local experts”, and all 25 of them willingly consented to participate. The following table contains general information about the participant profile.

Socio-economic descriptors of the profile of study participants:

| **Socio-economic descriptor** | **n** | **%** |
| --- | --- | --- |
| **Gender** |  |  |
| Female | 4 | 16 |
| Male | 21 | 84 |
| **Age range** |  |  |
| Less than 20 years | 0 | 0 |
| Between 20 and 30 years | 0 | 0 |
| Between 30 and 40 years | 4 | 16 |
| Between 40 and 50 years | 4 | 16 |
| More than 50 years | 17 | 68 |
| **Education level** |  |  |
| No formal education | 5 | 20 |
| Elementary school | 12 | 48 |
| High school | 8 | 32 |
| **Monthly income** |  |  |
| Less than US$ 280 | 2 | 8 |
| US$ 280 (Brazilian minimum wage) | 16 | 64 |
| More than US$300 | 3 | 12 |
| Not declared | 4 | 16 |
| **Main occupation** |  |  |
| Fisher (or shellfish collector) only | 13 | 52 |
| Fisher with a secondary occupation | 9 | 36 |
| Retired fisher | 3 | 12 |

*Workshops*

To complement the interviews, we organized workshops involving various estuary user groups to map significant areas and identify threats and management options. In December 2021, two workshops were held in municipalities located within the protected area (Tamandaré and Rio Formoso). Invitations to the workshop were sent to the fishermen's association, marina and protected areas administration. These organizations recommended key participants who volunteered to participate in the workshops. In total, we had the participation of 24 local actors, covering diverse groups, including small-scale fishers, boat operators, entrepreneurs, protected area managers and government environmental agencies, which are described below.

- Small-scale fishers: the associations indicated fishers who have been fishing throughout the estuary for many years and who have knowledge about the areas where seahorses occur, and the problems related to threats.
- Boa operators: one of the workshops convened at a marina in Tamandaré municipality, drawing significant participation from boat operators primarily engaged in tourism activities. Alongside these operators, business figures from this sector also participated in the workshop.
- Protected area managers and government environmental agencies: representing this sector, the managers of the Guadalupe Environmental Protection Area (Área de Proteção Ambiental de Guadalupe - APAG) and a representative of the environmental secretary of the municipality of Rio Formoso participated in the workshops.

**S2. Questionnaire** **to access fishers’ knowledge and to carry out the mapping of threats and seahorses’ distribution in the Rio Formoso Estuary.**

| **Participant profile** |
| --- |
| How old are you? |
| How long have you lived here? |
| What is your level of education? |
| What do you do for a living? |
| What is your monthly income? |
| Do you carry out another economic activity? Which? |
| Are you a fisher only or a boat conductor? (fisher only, boat conductor only, both) |
| In which part of the estuary do you fish? How long? |
| What type of fishing do you do? |
| Which type of gear/net do you use (mesh size)? |
| Do you do this type of fishing all year round? |
| Are you associated with any fisher’s colony or association? |
| **Biological information** (to check their knowledge on seahorses) |
| Have you ever seen a seahorse? |
| What type of environment do you find the most seahorses? |
| Do you know how to differentiate the male from the female? |
| Do seahorses live in freshwater or saltwater? |
| **Seahorse distribution** |
| In which part of the estuary are there seahorses? |
| Are there more or fewer seahorses here these days? If less, when was there more? Why is there less? |
| **Seahorse capture** |
| Have you ever caught a seahorse? If yes, for what purpose (intentional or unintentional)? |
| If yes, how often does this happen? |
| If yes, what do you do with captured seahorses? |
| **Seahorse conservation** |
| What do you think harms seahorses most here in the estuary? |
| **Mapping instructions** |
| Point out areas on the map where there are seahorses now (green) |
| Point on the map the areas where the threats you mentioned are concentrated (e.g., non-selective fishing, nautical traffic, pollution) |
| Point out on the map the areas that you think are most important to protect for seahorses here in the estuary (i.e., priority areas) |
| Point out on the map areas that hold cultural or socioeconomic significance to you (e.g., tourist attractions, economic activity zones, boat docking areas) |

**S3. Data used, processing, and details on conservation features mapping**

We used three primary sources of data: academic data collection methods, existing science-derived data, and local ecological knowledge.

*Academic data collection*

From December 2020 to July 2021, two of the authors conducted 125 transect surveys and extensive searches at 46 points across the estuary, covering a total area of 12,500 m2 (Figure S1). The primary purpose of this academic data collection was to conduct underwater visual surveys for seahorse distribution and to collect habitat data. Throughout this survey, the researchers also identified and documented spatial information regarding the occurrence of use activities and potential threats.

*Existing science-derived data*

We accessed existing science-derived spatial data products (which utilize remote sensed data and modelling procedures) on land cover and land use from the MapBiomas Project (https://mapbiomas.org/)

*Local Ecological Knowledge*

Interviews and focus groups were used to elicit LEK on the distribution and habitat of seahorses as well as threats to them. This resulted in LEK elicited spatial data on seahorse occurrences, priority areas, and threats.

Through surveys we identified the primary types of threats to seahorses which include water-based threats and land based threats. For water-based threats, participatory mapping was used to elicit spatial data on the occurrence and distribution of activities.

For land-based threats, survey participants identified three primary sources of diffuse pollution that impact the RFE: aquaculture (specifically shrimp farming), agriculture (involving the use of herbicides and insecticides), and urban pollution. They also identified that associated with urban areas there is point source pollution from the improper disposal of untreated sewage and waste. To map areas effected by each of these land-based pollution sources we utilized science derived remote sensed land cover data. Procedures are described below for each pollution source.

*Marxan data processing*

Here, we describe the procedures used to derive the distribution of the conservation features included in the prioritization scenarios. We detail each layer processing below and annotate the layers as primarily science derived or LEK derived.

Marxan requires the “Planning Unit versus Conservation Resources File”, which contains information about the distribution of conservation resources in each of the planning units. To calculate this, we use the tools available at CLUZ interface (http://www.kent.ac.uk/dice/cluz/). All procedures described were carried out using QGIS version 3.32.2. Our methodology adhered to the data preparation and analysis guidelines outlined in the Marxan Good Practices Handbook (Ardron et al., 2010).

*Planning area and planning units*

The planning area is the Rio Formoso Estuary (RFE), the extent of which was mapped in this study resulting in a shapefile of the area. Subsequently, the planning area extension shapefile was used to generate the planning units (PUs) file using the Vector -> Search Tools -> Create Grid function in QGIS. We used a hexagonal shape for the PUs and a size of 250m², resulting in 3710 PUs.

*Seahorse abundance (science-derived)*

The seahorse abundance data used in the science-derived abundance scenario and the four threat management scenarios were derived from a prior survey of the area conducted by Borges et al. (2023). This comprehensive mapping effort assessed seahorse abundance throughout the Rio Formoso Estuary (Figure S1). Abundance data were collected through visual surveys to assess the distribution and density of *Hippocampus reidi* in the Rio Formoso Estuary using random linear transects (50 × 2 m). Using a randomly stratified approach, 125 transect surveys were carried out across the estuary, surveying a total of 12,500 m². Transect densities were converted to a continuous spatial layer with a kernel density function. More details about the methodology used by the study to collect this data can be accessed in Borges et al. (2023). Using the seahorse abundance database from this study, we calculated seahorse abundance within each PU to produce the spatial dataset for this conservation feature, which served as input data for the Marxan prioritization process.

*Seahorse presence (LEK-derived)*

The seahorse distribution data employed in the LEK-derived scenario originated from the participatory mapping exercises carried out as part of this study. Participants, deliberately selected because they are "local experts" who have vast knowledge about the estuary and seahorses, marked seahorse occurrence points on a base map, and these maps were subsequently overlaid to produce a comprehensive seahorse distribution map for the RFE based on Local Ecological Knowledge (Figure S2). To generate the Marxan input data for this conservation feature, we overlap the distribution map onto the planning unit (PU) layer and calculated the number of mentions provided by the informants for each PU. This process facilitated the incorporation of LEK into the Marxan prioritization analysis.

*Mangrove coverage (Science-derived)*

As emphasized by Borges et al. (2023), the presence and extent of mangrove coverage stand out as the most influential factors affecting seahorse occurrence in the RFE. Consequently, we utilized this feature as a proxy for seahorse habitat protection, primarily because comprehensive mapping for other habitat types in the region is unavailable. We acquired refined-resolution (10m) data on mangrove coverage within the RFE from the sixth dataset of the open-source MapBiomas Project (https://mapbiomas.org/; Figure S3). To obtain information on the distribution of this conservation feature to Marxan prioritization, we computed the area (in square meters) covered by mangroves within each PU.

*Non-selective fishing (LEK-derived)*

During the participatory mapping exercises, participants identified areas within the RFE where non-selective fishing gear that directly threatened seahorses was employed. This dataset was further enriched with information gathered by the authors in prior studies conducted in the region. The resulting maps were then merged to create a comprehensive map (Figure S4). Subsequently, this map and the PU layer were overlapped to generate a binary input for Marxan. In this binary map, a value of 1 indicated the presence of the non-selective fishing activity within each PU, while 0 signified its absence.

*Nautical traffic (LEK-derived)*

The procedure used to create the mapping of areas primarily impacted by nautical traffic closely resembled the process outlined for the non-selective fishing map (Figure S5). In this instance, we supplemented the data obtained from participatory mapping exercises with information sourced from the report of the Environmental and Territorial Zoning of Nautical Activities (ZATAN) of the RFE (<https://www.bivica.org/file/view/id/5963>).

*Agriculture (Science-derived)*

To obtain a proxy of the RFE areas affected by agriculture, we utilized land cover and land use maps sourced from the MapBiomas collection. The raster data were confined to the study area's boundaries, and solely the "Agriculture" feature (code 3.2.) was considered (Predominantly depicting the extent of sugarcane cultivation). We applied a 1 km buffer to the agricultural coverage areas (Figure S6a) and then overlaid the map with the PU layer. We apply this buffer size because it has been used in other studies to delineate the extent of terrestrial threats in the absence of more accurate data (Berger et al., 2022; Temino-Boes et al., 2020). To create the Marxan input data, PUs falling within the 1 km buffer were designated as 1, signifying the presence of the agricultural threat, while those outside the buffer were marked as 0, indicating the absence of this threat.

*Aquaculture (Science-derived)*

The process applied was very similar to the process described for the agriculture threat layer. Here, we used the "Aquaculture" feature (code 5.2) from the MapBiomas collection, which represents the areas covered by shrimp farms on the RFE (Figure S6b).

*Urban areas (Science-derived)*

The procedure employed closely mirrored the one described for the agriculture threat layer. For this layer, we used the "Urban Area" feature (code 4.2) from the MapBiomas collection, which represents the urbanized areas near the RFE (Figure S6d). We used this data since urban areas are considered the primary source of waste disposal.

*Sewage discharge (point source pollution) (Science-derived)*

The procedure employed closely mirrored the one described for the agriculture threat layer. In this case, we utilized data points indicating the locations where untreated sewage is discharged, as reported by survey participants (Figure S6c).

*Important socioeconomic spots (LEK-derived)*

Throughout the participatory mapping exercises, participants were asked to pinpoint areas within the RFE that held invaluable socioeconomic and historical significance, including commercial hubs, tourist destinations, ports, and historical landmarks. This information was also complemented by the author's knowledge of the area to create a map of socioeconomic important sites on the RFE (Figure S7). This map was used to lock PUs within these sites, preventing their inclusion in the Marxan solutions to safeguard their significance for the local community.

**S4. Marxan parameters calibration**

Setting appropriate values for SPF and BLM

In Marxan, the Boundary Length Modifier (BLM) is a parameter used to regulate the compactness of selected planning units within a conservation area (Ardron et al., 2010; Ball et al., 2009; Watts et al., 2009). It helps manage the spatial arrangement of selected units to maintain a more ecologically connected and practical configuration. The BLM value influences the trade-off between spatial compactness and achieving conservation objectives.

The Species Penalty Factor (SPF) in Marxan is a parameter used to regulate the costs associated with the inclusion of planning units that host specific species or biodiversity features (Ardron et al., 2010; Ball et al., 2009; Watts et al., 2009). It helps balance the selection of planning units to represent different species or biodiversity targets while considering the costs of including those units in the final solution. A higher SPF value indicates a higher cost or penalty for incorporating planning units that are vital for the conservation of specific species or features. This parameter aids in prioritizing planning units that are essential for achieving conservation objectives. Adjusting the SPF allows planners to control the influence of species or features on the overall conservation solution while considering the associated costs.

An iterative approach was employed to determine suitable SPF values and BLM. The values were calibrated through an iterative manual calibration method, adhering to the guidelines set in the MARXAN good practices handbook and as described in previous studies (Ardron et al., 2010; Zhang & Vincent, 2019). To achieve a satisfactory inclusion of the biodiversity features within a spatially efficient and ecologically connected layout in all the scenarios, through the iterative process we set a BLM value of 4 and different SPF values for each feature (seahorse distribution = 10, mangrove cover = 5, threats = 1).

**S5. LEK-Derived spatial priorities**

*Seahorse Priority areas (LEK-derived)*

During the participatory mapping exercises, participants were asked to identify areas they deemed essential for seahorse conservation. Those maps were combined according to the frequency of mentions per site. Subsequently, we employed the Kernel Density tool to generate a density map that delineated the priority areas for seahorse conservation according to LEK (Figure S8).


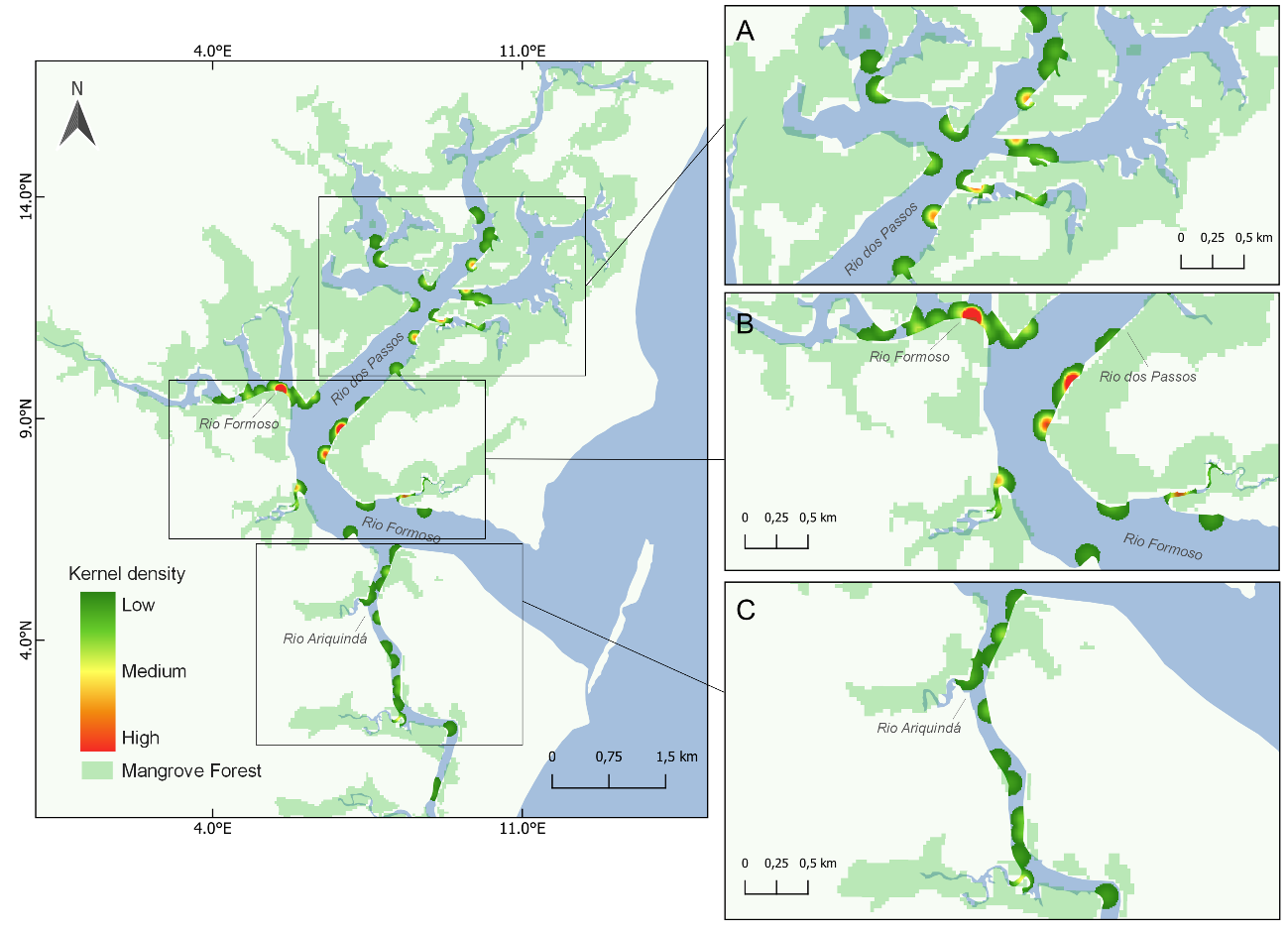


**Figure S1.** Seahorse distribution in the Rio Formoso Estuary (Pernambuco, Brazil). Source: Borges et al. (2023).


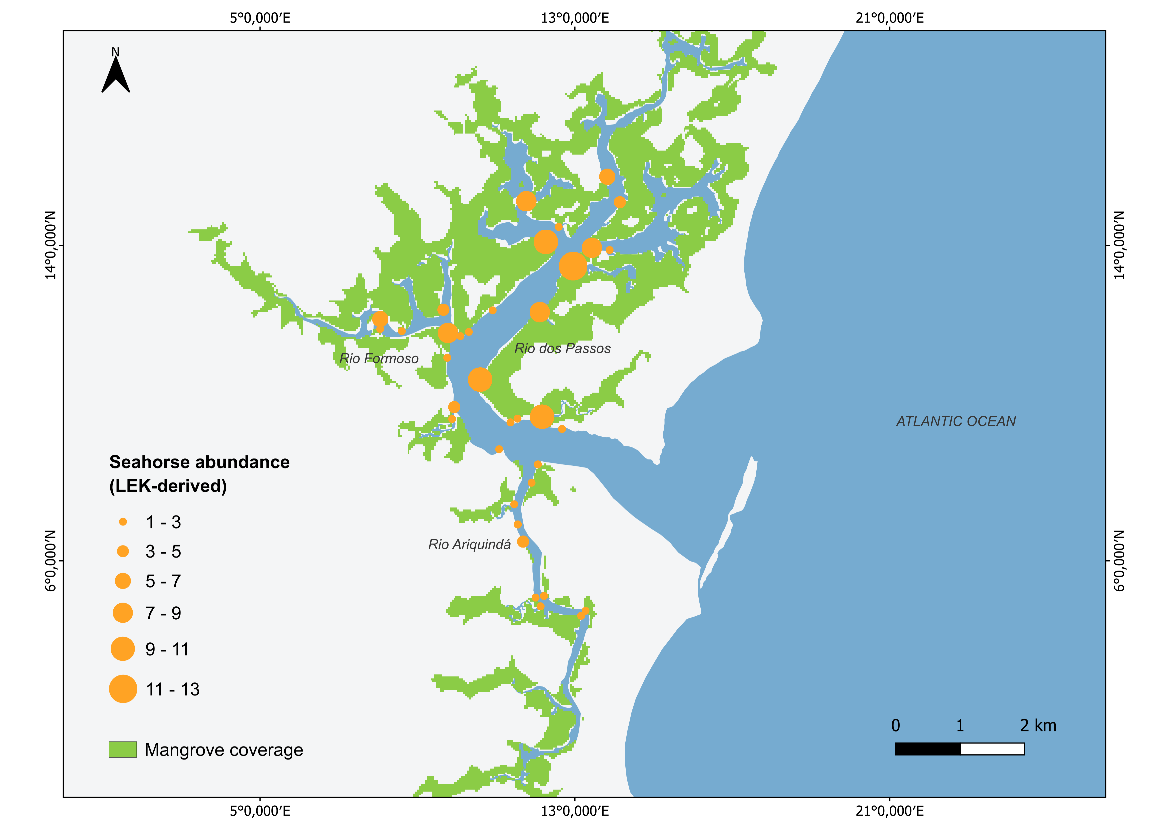


**Figure S2.** LEK-derived seahorse distribution in the Rio Formoso Estuary (Pernambuco, Brazil).


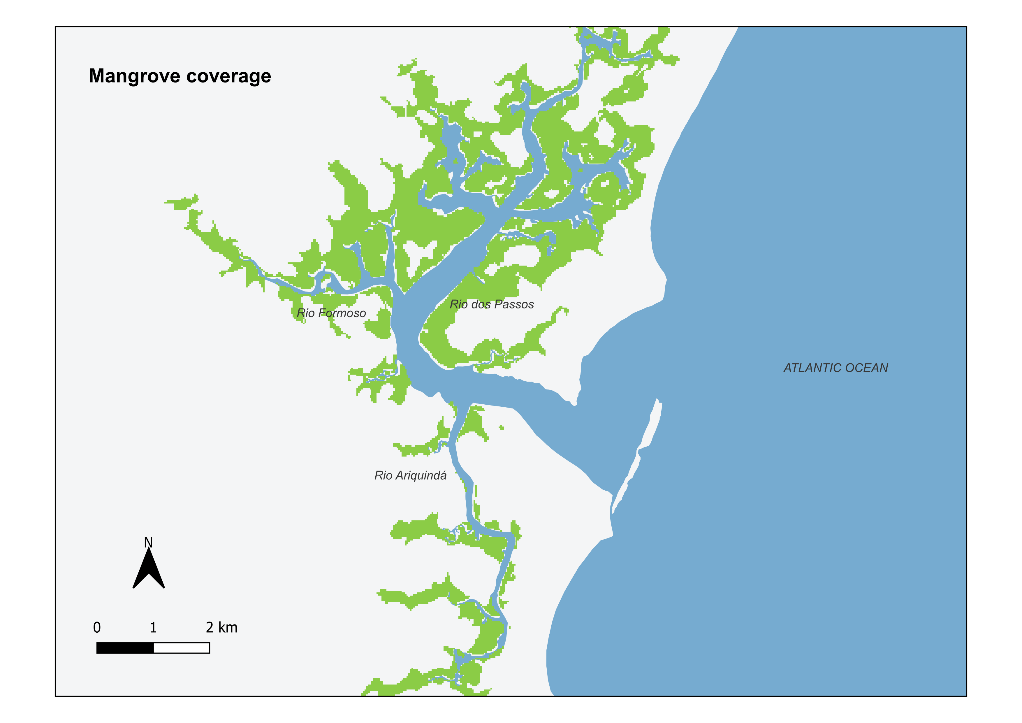


**Figure S3.** Mangrove coverage in the Rio Formoso Estuary (Pernambuco, Brazil) according to 2022 data from the MapBiomas Project (available in <https://mapbiomas.org/>).


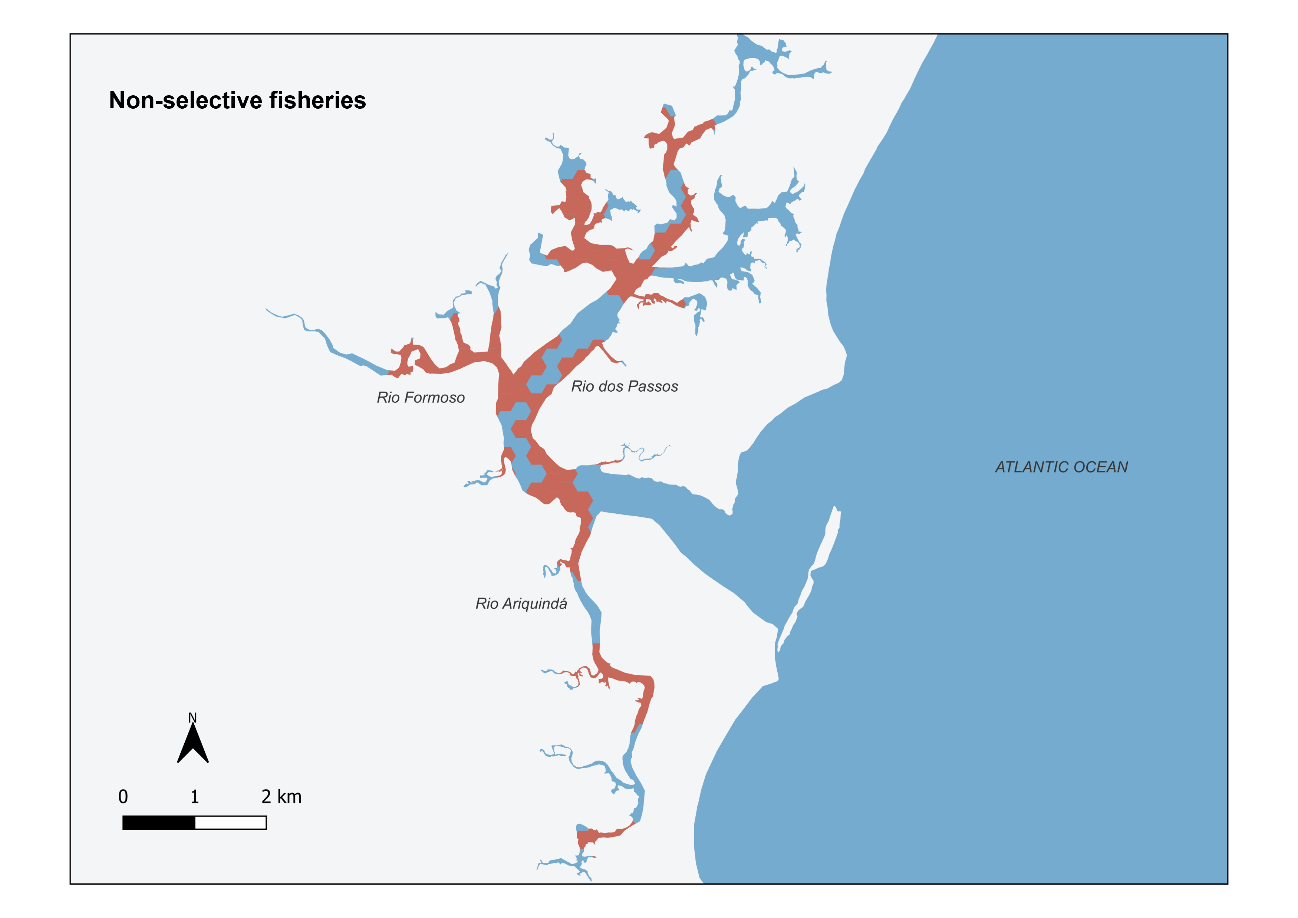


**Figure S4.** Map of non-selective fishing threatening seahorses in the Rio Formoso Estuary.


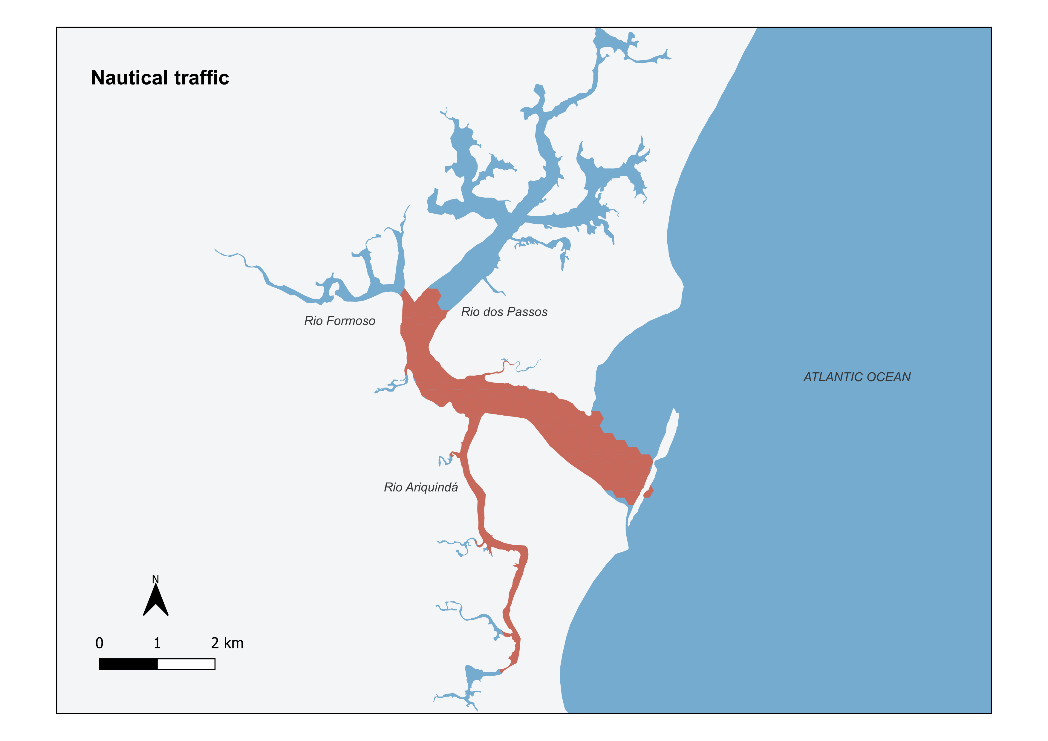


**Figure S5.** Map of areas with high-intensity nautical traffic in the Rio Formoso Estuary.


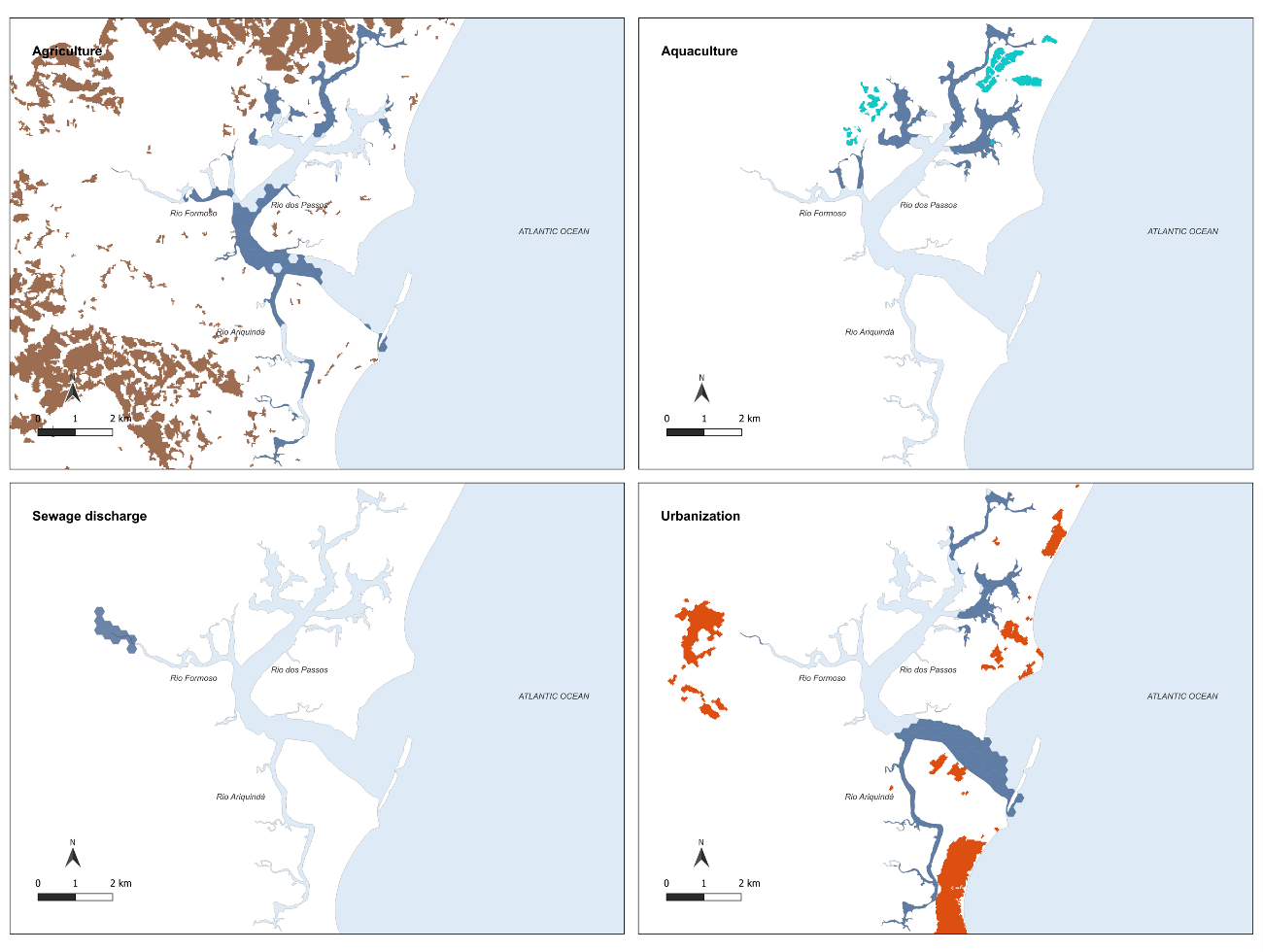


**Figure S6**. Map of land-based threats on the Rio Formoso Estuary. Respectively: agriculture, aquaculture (shrimp farm), non-treated sewage discharge, and urbanized areas.


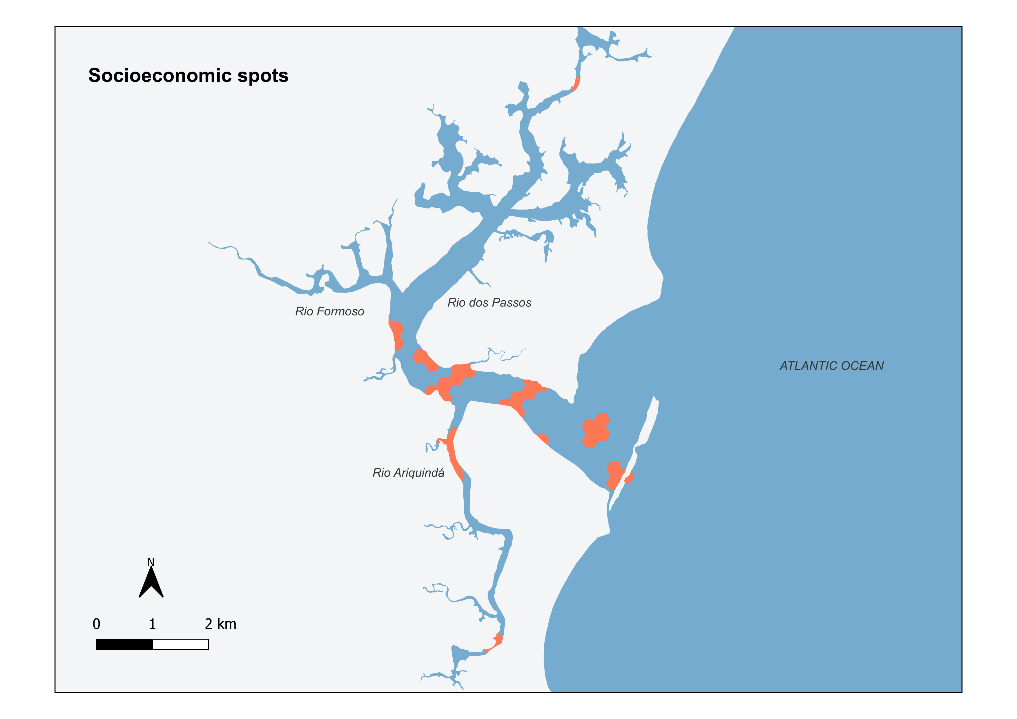


**Figure S7.** Map of sites with high socioeconomic and historical importance for the local community in the Rio Formoso Estuary.


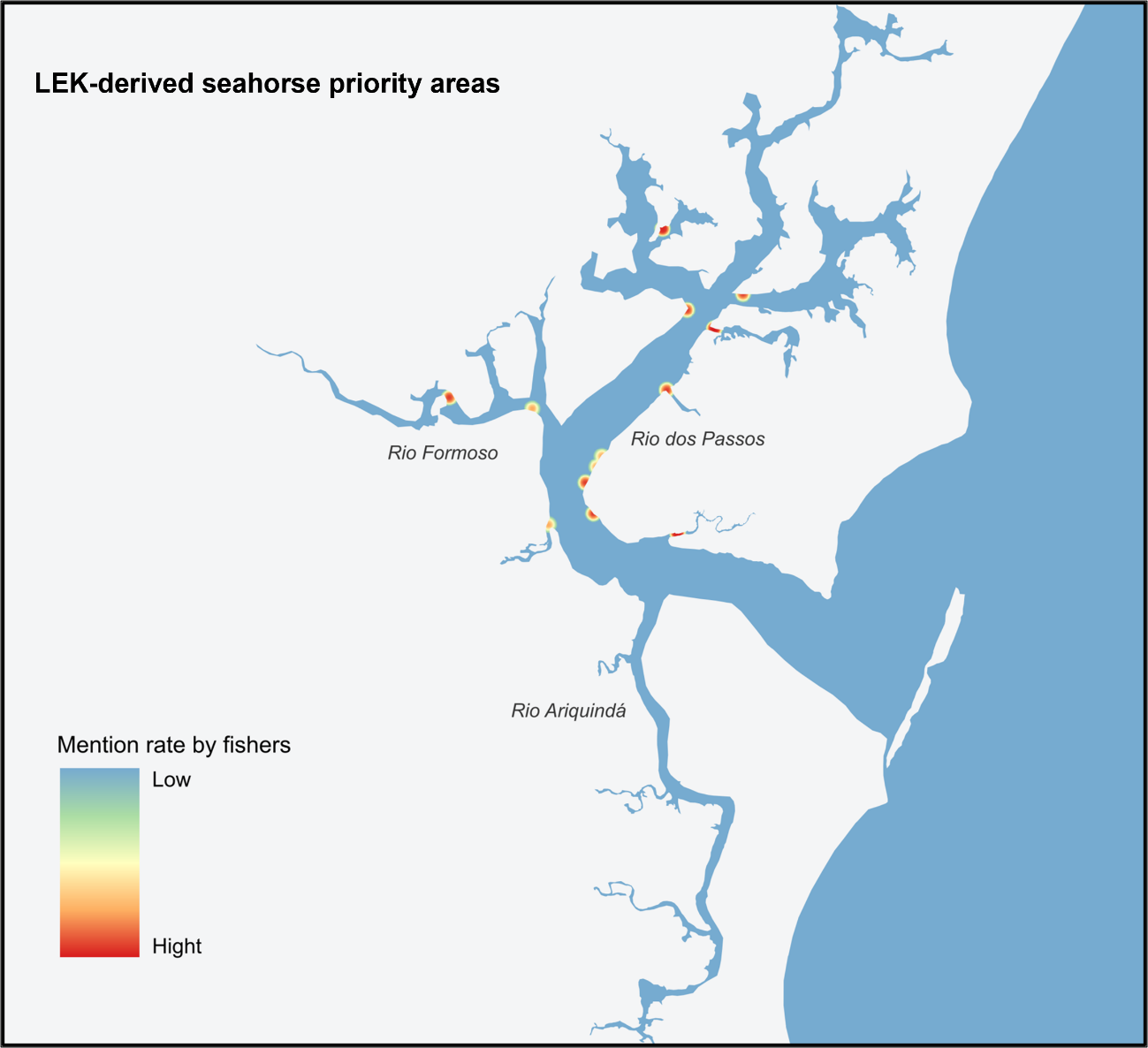


**Figure S8.** Priority areas for seahorse conservation in the Rio Formoso Estuary informed by Local Ecological Knowledge (LEK).

**REFERENCES**

Ardron, J.A., Possingham, H.P., and Klein, C.J. (2010). *Marxan Good Practices Handbook, Version 2.* Pacific Marine Analysis and Research Association, Victoria, BC, Canada.

Ball, I. R., Possingham, H. P., & Watts, M. (2009). *Marxan and relatives: software for spatial conservation prioritisation*. In: Spatial conservation prioritisation: quantitative methods and computational tools. Oxford University Press, Oxford, 185-196.

Berger, M., Canty, S. W., Tuholske, C., & Halpern, B. S. (2022). Sources and discharge of nitrogen pollution from agriculture and wastewater in the Mesoamerican Reef region. *Ocean & Coastal Management*, 227, 106269.

Borges, A. K. M., Alves, R. R. N., & Oliveira, T. P. R. (2023). Mapping seahorses in a Brazilian estuary: mangrove structures as key predictors for distribution and habitat preference. *PeerJ*, 11, e15730.

Temino-Boes, R., Romero-Lopez, R., Ibarra-Zavaleta, S. P., & Romero, I. (2020). Using grey clustering to evaluate nitrogen pollution in estuaries with limited data. *Science of the Total Environment*, 722, 137964.

Watts, M. E., Ball, I. R., Stewart, R. S., Klein, C. J., Wilson, K., Steinback, C., ... & Possingham, H. P. (2009). Marxan with Zones: Software for optimal conservation based land-and sea-use zoning. *Environmental Modelling & Software*, 24(12), 1513-1521.

Zhang, X., & Vincent, A. C. (2019). Conservation prioritization for seahorses (Hippocampus spp.) at broad spatial scales considering socioeconomic costs. *Biological Conservation*, 235, 79-88.
